# Comparison of the effects of transcranial direct current stimulation combined with different rehabilitation interventions on motor function in people suffering from stroke-related symptoms: a systematic review and network meta-analysis

**DOI:** 10.3389/fneur.2025.1586685

**Published:** 2025-06-04

**Authors:** Kaiqi Zheng, Liang Guo, Weidong Liang, Pengcheng Liu

**Affiliations:** School of Physical Education and Sports Science, South China Normal University, Guangzhou, Guangdong, China

**Keywords:** stroke, network meta-analysis, motor function, upper limb, transcranial direct current stimulation

## Abstract

**Background:**

This study employs network meta-analysis to assess the efficacy of transcranial direct current stimulation (tDCS) combined with different rehabilitation approaches in enhancing motor function in people suffering from stroke-related symptoms (PSSS). The objective is to determine the most effective tDCS-based rehabilitation approach and offer valuable evidence to guide clinical decision-making.

**Methods:**

This study included randomized controlled trials (RCTs) published before September 23, 2024. We conducted a systematic search across eight databases: PubMed, Embase, Cochrane Library, Web of Science, China National Knowledge Infrastructure (CNKI), China Biology Medicine (SinoMed), Wanfang, and VIP. Network meta-analysis (NMA) was conducted utilizing R Studio and Stata 15.0 for data analysis.

**Results:**

A total of 74 RCTs were included in this study, encompassing 4,335 PSSS and 11 intervention strategies. The NMA revealed that brain-computer interface therapy (BCIT) in combination with tDCS [surface under the cumulative ranking curve (SUCRA) = 88.34%] was the most effective tDCS-based intervention for improving the Fugl-Meyer Assessment for Upper Extremity score in PSSS. Mirror therapy (MT) in combination with tDCS (SUCRA = 85.96%) was identified as the optimal intervention for enhancing the Action Research Arm Test score in PSSS. MT + tDCS (SUCRA = 84.29%) was the best approach for improving the Fugl-Meyer Assessment for Lower Extremity score. Additionally, acupuncture and moxibustion (AM) in combination with tDCS (SUCRA = 77.16%) was the most effective intervention for increasing the Berg Balance Scale score in PSSS. The two-dimensional clustering analysis showed that MT + tDCS (SUCRA = 75.83%/85.96%) was the optimal tDCS-based rehabilitation strategy for treating upper limb motor dysfunction in PSSS, while AM+tDCS (SUCRA = 76.94%/77.16%) was the best tDCS-based rehabilitation strategy for improving lower limb motor dysfunction in PSSS.

**Conclusion:**

BCIT+tDCS was identified as the optimal tDCS-based rehabilitation strategy for improving upper limb motor ability in PSSS, MT + tDCS was the most effective intervention for enhancing arm mobility, MT + tDCS was the best protocol for improving lower limb motor ability, while AM+tDCS was the best strategy for improving balance ability. Furthermore, MT + tDCS was the optimal tDCS-based rehabilitation approach for treating upper limb motor dysfunction, whereas AM+tDCS was the most effective strategy for addressing lower limb motor dysfunction in PSSS. Future studies may focus on investigating the therapeutic effects of MT combined with tDCS on Berg Balance Scale score in PSSS, as well as the effects of AM combined with tDCS on Action Research Arm Test score, in order to further explore the therapeutic potential of these two intervention strategies.

**Systematic review registration:**

https://www.crd.york.ac.uk/PROSPERO/view/CRD42024621998, Identifier PROSPERO CRD42024621998.

## 1 Introduction

With population aging and lifestyle changes, the number of stroke cases has been increasing annually (1). It has become the world’s third leading cause of disability and the second most common cause of death among adults (2). The high disability rate associated with stroke significantly impacts patients’ quality of life, as many stroke survivors experience varying degrees of motor dysfunction, including marked weakness in the affected limbs, restricted joint range of motion, impaired balance, and coordination deficits (3, 4). Currently, the rehabilitation of motor dysfunction in people suffering from stroke-related symptoms (PSSS) remains a major challenge in clinical practice.

Current research suggests that a range of rehabilitation interventions, including acupuncture and moxibustion, functional electrical stimulation, core stability training, proprioceptive training, and transcranial direct current stimulation (tDCS), are widely employed to manage motor dysfunction in individuals with stroke-related symptoms (PSSS), aiming to enhance motor recovery and functional mobility (5–9). Among these, tDCS has demonstrated particularly promising therapeutic effects (10). tDCS is a non-invasive neuromodulation technique (11) that enhances excitability in the affected cortical region, reduces inhibitory effects from the healthy hemisphere, and improves local cerebral blood flow to protect neurons in ischemic areas (12). Based on these mechanisms, numerous clinical studies have confirmed the effectiveness of tDCS in treating motor dysfunction in PSSS. For example, Lindenberg et al. (13) reported that PSSS receiving tDCS exhibited greater improvements in upper limb motor function compared to those receiving sham tDCS. Similarly, Cha et al. (14) found that tDCS treatment resulted in significantly greater improvements in both upper and lower limb function in PSSS than in those who did not receive tDCS. Over the past two decades, the clinical application of tDCS in PSSS motor rehabilitation has matured, with most studies reporting favorable outcomes. To further enhance the effectiveness of motor rehabilitation in PSSS, researchers have explored the combination of tDCS with other rehabilitation techniques. Tedla et al. (15) found that PSSS receiving tDCS combined with proprioceptive training exhibited significantly greater improvements in upper limb motor ability compared to those receiving sham tDCS. Additionally, a study by Cui et al. (16) demonstrated that tDCS combined with acupuncture and moxibustion led to significantly greater improvements in balance ability than conventional rehabilitation. Compared to standalone tDCS or conventional rehabilitation, combined tDCS-based therapies have consistently shown superior efficacy in motor function rehabilitation for PSSS.

Network meta-analysis (NMA) is a specialized form of meta-analysis that integrates direct, indirect, and mixed comparisons of different interventions. This approach enables the evaluation of the relative efficacy of various treatments and ultimately facilitates the identification of the most effective intervention (17). In this study, we applied NMA to compare the therapeutic effects of tDCS combined with other rehabilitation interventions in the rehabilitation of motor dysfunction in PSSS. The objective is to determine the most effective tDCS-based rehabilitation approach and offer valuable evidence to guide clinical decision-making.

## 2 Methods

This network meta-analysis (NMA) was performed following the 2020 Preferred Reporting Items for Systematic Reviews and Meta-Analyses (PRISMA) guidelines and the methodological framework of the Cochrane Handbook (18). Additionally, this study has been registered in PROSPERO under the registration number CRD42024621998.

### 2.1 Search strategy

An extensive electronic literature search was conducted across eight databases: PubMed, Embase, Cochrane Library, Web of Science, CNKI, SinoMed, WanFang, and VIP, covering studies published until September 23, 2024. Tailored search strategies were applied to meet the specific criteria of each database, with detailed methodologies available in Supplementary Table 1.

### 2.2 Inclusion criteria

(1) Participants: studies included adult PSSS (people suffering from stroke-related symptoms) aged ≥18 years who met the diagnostic criteria for stroke and were confirmed to have motor function impairment. (2) Intervention in the Experimental Group: Participants received tDCS or tDCS combined with other rehabilitation therapies, with conventional rehabilitation as the baseline treatment. (3) Intervention in the control group: participants received sham stimulation or conventional rehabilitation. (4) Outcome measures: Fugl-Meyer Assessment-Upper Extremity (FMA-UE): Evaluates upper limb motor ability, with higher scores indicating better motor function. Action Research Arm Test (ARAT): Assesses arm movement ability, with higher scores reflecting better arm mobility. Fugl-Meyer Assessment-Lower Extremity (FMA-LE): Measures lower limb motor ability, where higher scores represent better lower limb function. Berg Balance Scale (BBS): Evaluates balance ability, with higher scores indicating better balance control. This study primarily focuses on NMA, which only requires included studies to report sufficient data on primary outcomes (BBS, FMA-LE, FMA-UE, ARAT). (5) Study design: randomized controlled trials (RCTs).

Due to the presence of certain heterogeneity in the original studies, there were no fully consistent standards regarding electrode placement, stimulation intensity, duration, and rehabilitation training protocols. During data extraction, we made every effort to record these key details for subsequent discussion of potential sources of heterogeneity. However, in the overall analysis, tDCS was treated as a unified intervention approach. Detailed tDCS stimulation parameters for all included studies are summarized in Supplementary Table 2.

### 2.3 Exclusion criteria

(1) Guidelines, conference abstracts, systematic review, meta-analyses, etc. (2) non-RCTs. (3) Duplicate publications. (4) Studies with interventions or outcome measures that do not meet the inclusion criteria, studies with significant data errors, or studies where data could not be obtained. (5) tDCS combination interventions with fewer than two eligible studies.

### 2.4 Study selection and data extraction

Three researchers jointly established the inclusion criteria for the studies. After removing duplicate records using EndNote X9 software, two researchers independently conducted the study selection process. Initially, screening was performed based on titles, keywords, and abstracts, followed by a full-text review for data extraction and verification. In cases of disagreement, a third researcher was consulted to resolve discrepancies until a consensus was reached. The extracted baseline data included the following: basic study information (authors, publication year), patient characteristics (age, sample size, disease duration), intervention details (treatment and control group interventions), and outcome measures.

### 2.5 Quality assessment

Two researchers independently evaluated the methodological quality of the included studies using the Risk of Bias 2.0 (ROB 2.0) tool, as detailed in the Cochrane Handbook. The evaluation results were cross-checked, and any disagreements were resolved through discussion with a third researcher until a consensus was reached. The assessment covered several domains, including the randomization process, deviations from intended interventions, completeness of outcome data, outcome measurement, and selective reporting bias. Each domain comprised 1–7 specific items. The overall risk of bias for each study was classified as “high risk,” “low risk,” or “some concerns (moderate risk).”

### 2.6 Data analysis

This study utilized R 4.2.1 with the “gemtc” and “coda” packages and Stata 15 with the “gemtc” package to perform NMA. Continuous outcome variables were analyzed using mean difference (MD), with 95% credible interval (CrI) for interval estimation. A consistency model was established with 20,000 burn-ins and 50,000 iterations. The deviance information criterion (DIC) was used to compare the consistency model with the inconsistency model, and if the DIC difference was <5, the direct and indirect comparisons were considered consistent. For each outcome measure, the surface under the cumulative ranking curve (SUCRA) was used to rank the efficacy of different interventions. A two-dimensional clustering analysis was conducted to explore the optimal intervention. A comparison-adjusted funnel plot was generated to assess potential publication bias. A regression analysis between publication year and intervention effect was conducted using R Studio to evaluate whether the publication year influences the intervention effect. This NMA employed a burn-in of 20,000 iterations and 50,000 subsequent iterations. Convergence diagnostics, including the Gelman-Rubin statistic and visual inspection of trace plots, confirmed that the model met the stability requirements.

## 3 Results

### 3.1 Literature search results

**Figure 1:**
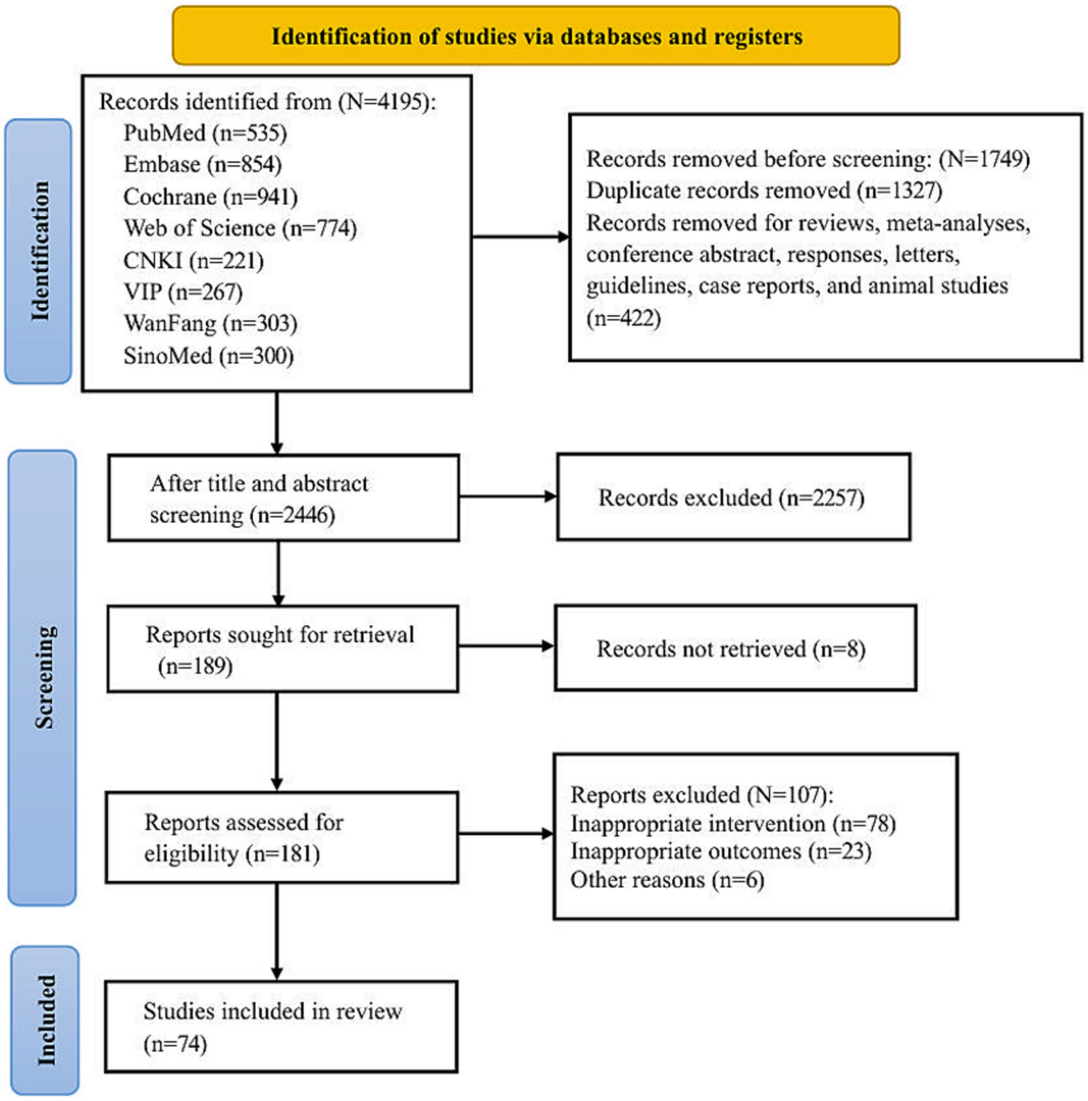
Flow chart of literature screening.

A total of 4,195 records were initially identified through the search strategy. After removing 1,327 duplicate records and excluding 422 records consisting of reviews, animal studies, non-randomized controlled trials (RCTs), and conference abstracts, 2,446 records remained. Title and abstract screening led to the exclusion of another 2,257 records. Of the 189 remaining studies, 8 lacked full-text access, 78 had inappropriate interventions, 23 had unsuitable outcome measures, and 6 were excluded due to other reasons (e.g., apparent data errors or unavailability of original data). Ultimately, 74 RCTs (13–16, 19–88) were included in this study. The process of study selection is depicted in Figure 1.

### 3.2 Basic characteristics of the included studies

A total of 74 RCTs were included in this study, involving 4,335 PSSS (people suffering from stroke-related symptoms) and 11 intervention strategies. Both the treatment and control groups received conventional rehabilitation (C) as the baseline therapy. In addition to transcranial direct current stimulation (tDCS), the treatment group included nine combined tDCS interventions: tDCS+MIT (motor imagery therapy), tDCS+MT (mirror therapy), tDCS+AM (acupuncture and moxibustion), tDCS+CIMT (constraint-induced movement therapy), tDCS+BCIT (brain-computer interface therapy), tDCS+VRT (virtual reality technology), tDCS+RRT (robotic rehabilitation therapy), tDCS+PT (proprioceptive training), and tDCS+FES (functional electrical stimulation). Table 1 provides an overview of the basic characteristics of the included studies.

**Table 1:** The basic characteristics of the included studies.

No.	Author	Year	Age (TG)	Age (CG)	N (TG)	N (CG)	Treatment (TG)	Treatment (CG)	Outcome	Duration (TG)	Duration (CG)
1	Che XW (19)	2017	61.49 ± 10.51	58.77 ± 14.3	41	39	MIT + tDCS	tDCS	FMA-UE	NR	NR
2	Chen HB (20)	2021	48.4 ± 13.2	50.1 ± 11.5	19	18	FES + tDCS	tDCS	FMA-LE BBS	0-6 m	0-6 m
3	Chen H (21)	2020	59.32 ± 8.59	61.31 ± 9.13	26	26	MT + tDCS	tDCS	FMA-UE	58.92 ± 17.30d	62.81 ± 18.14d
4	Chen TT (22)	2023	56.45 ± 9.57	55.10 ± 10.18	20	20	FES + tDCS	tDCS	FMA-UE	28.85 ± 4.65d	28.15 ± 5.13d
5	Chen Y (23)	2022	62.03 ± 8.45	62.84 ± 8.53	55	55	MT + tDCS	tDCS	FMA-UE FMA-LE	3.46 ± 0.81 m	3.42 ± 0.76 m
6	Cheng P (24)	2015	61.08 ± 7.34	57.27 ± 6.87	19	19	tDCS	C	FMA-UE	51.12 ± 19.4d	48 ± 16.45d
7	Cheng XX (25)	2024	61.59 ± 10.97	62.57 ± 12.70	13	11	tDCS	C	FMA-UE	33.23 ± 5.86w	32.45 ± 5.39w
8	Cui C (16)	2021	64.87 ± 5.59	65.27 ± 5.14	28	27	AM+tDCS	C	BBS	6.47 ± 1.20w	6.57 ± 1.20w
9	Deng R (26)	2024	67.82 ± 6.80	61.24 ± 7.43	43	43	tDCS	C	FMA-UE	NR	NR
10	Dong K (27)	2021	54.10 ± 7.31	52.30 ± 5.03	10	10	tDCS	C	BBS	31.80 ± 5.55d	32.20 ± 14.02d
11	Feng CW (28)	2023	58.39 ± 9.12	56.29 ± 6.23	28	28	CIMT+tDCS	C	FMA-UE	57.79 ± 32.61d	52.93 ± 26.02d
12	Gao L (29)	2024	55.73 ± 8.60	54.60 ± 8.23	15	15	BCIT+tDCS	tDCS	FMA-UE ARAT	58.47 ± 30.98d	70.27 ± 46.49d
13	Gao Z (30)	2021	55.8 ± 10.9	56.1 ± 10.7	45	45	MIT + tDCS	tDCS	FMA-UE	33.6 ± 12.9d	33.9 ± 13.3d
14	Gao Z (31)	2023	71.18 ± 5.13	71.17 ± 5.15	71	71	MT + tDCS	tDCS	FMA-UE	<3 m	<3 m
15	Han X (32)	2023	67.51 ± 10.72	67.04 ± 10.73	47	49	tDCS	C	FMA-UE	39.57 ± 22.25d	34.27 ± 19.78d
16	Hu HL (33)	2023	61.45 ± 1.63	60.23 ± 1.74	40	40	tDCS	C	FMA-UE	36.41 ± 1.72d	35.23 ± 1.41d
17	Huang Y (34)	2023	42.67 ± 15.62	43.43 ± 14.57	50	50	tDCS	C	FMA-UE	NR	NR
18	Jiang Y (35)	2020	53.19 ± 5.95	52.35 ± 6.03	15	15	tDCS	C	FMA-UE	21.84 ± 13.81d	22.09 ± 12.05d
19	Jin J (36)	2019	53.1 ± 5.3	52.2 ± 5.2	45	45	tDCS	C	FMA-UE ARAT	36.1 ± 3.8d	35.5 ± 5.7d
20	Jin MY (37)	2020	62 ± 5.23	62 ± 5.34	30	30	tDCS	C	FMA-LE BBS	1.5 ± 1.02	1.5 ± 1.04
21	Li XL (38)	2021	50.37 ± 14.03	52.43 ± 15.12	162	162	MT + tDCS	tDCS	FMA-UE ARAT	76.06 ± 20.95d	72.13 ± 21.18d
22	Li YB (39)	2019	52.43 ± 12.15	50.12 ± 11.35	14	15	MT + tDCS	tDCS	FMA-UE ARAT	72.11 ± 22.19d	75.02 ± 18.17d
23	Liu LS (40)	2019	48.39 ± 11.2	48.39 ± 11.2	33	32	tDCS	C	FMA-UE ARAT	1-6 m	1-6 m
24	Liu Y (41)	2023	58.83 ± 5.89	58.76 ± 5.62	47	46	MT + tDCS	tDCS	FMA-UE	52.71 ± 11.23d	53.35 ± 10.85d
25	Liu YW (42)	2020	62.86 ± 9.05	63.53 ± 7.26	15	15	VRT + tDCS	tDCS	FMA-UE	9.33 ± 2.15 m	9.67 ± 2.26 m
26	Long SY (43)	2024	54.35 ± 6.02	55.47 ± 5.53	55	55	tDCS	C	FMA-UE FMA-LE	NR	NR
27	Pan AH (44)	2023	68.27 ± 7.83	67.95 ± 7.96	50	50	CIMT+tDCS	tDCS	FMA-UE ARAT	51.67 ± 13.89d	49.29 ± 14.12d
28	Qi YS (45)	2023	63.25 ± 2.21	63.18 ± 2.13	35	35	RRT + tDCS	C	FMA-UE FMA-LE	3.21 ± 0.24 m	3.29 ± 0.25 m
29	Qu F (46)	2024	59	63	30	30	AM+tDCS	tDCS	FMA-UE	59d	47d
30	Ren SS (47)	2023	66.18 ± 3.98	66.21 ± 4.06	28	28	MIT + tDCS	tDCS	FMA-UE	62.86 ± 12.67d	56.43 ± 10.18d
31	Song DW (48)	2024	62.01 ± 1.87	61.49 ± 1.83	41	41	tDCS	C	ARAT	NR	NR
32	Sun FB (49)	2023	62.15 ± 9.40	61.35 ± 9.82	20	20	tDCS	C	FMA-UE	37.95 ± 5.07d	38.10 ± 4.75d
33	Tu M (50)	2021	64.2 ± 6.9	63.5 ± 8.0	75	75	tDCS	C	FMA-UE	NR	NR
34	Wang C (51)	2021	61.39 ± 6.52	61.20 ± 6.31	54	53	tDCS	C	FMA-UE	5.77 ± 0.27 m	5.61 ± 0.48 m
35	Wang CY (52)	2023	62.80 ± 11.16	62.67 ± 10.70	15	15	BCIT+tDCS	tDCS	ARAT	13.60 ± 2.16d	12.53 ± 2.59
				59.13 ± 11.93		15		C			13.93 ± 2.84
36	Wang HB (53)	2023	57.24 ± 8.23	57.81 ± 9.22	20	20	CIMT+tDCS	tDCS	FMA-LE BBS	35.62 ± 32.14d	37.22 ± 31.16d
37	Wang HY (54)	2023	60.25 ± 5.84	54.07 ± 6.15	40	40	AM+tDCS	tDCS	FMA-UE	1.15 ± 0.78 m	1.30 ± 0.71 m
38	Wang Y (55)	2021	60.95 ± 9.31	58.95 ± 7.2	19	20	AM+tDCS	tDCS	FMA-UE	1.2 ± 0.66 m	1.22 ± 0.74 m
39	Yin Y (56)	2015	55.70 ± 12.32	57.68 ± 13.54	40	40	tDCS	C	FMA-UE ARAT	31.55 ± 20.13d	35.90 ± 19.60d
40	Zhang SS (57)	2022	61.83 ± 6.29	61.26 ± 5.29	46	46	AM+tDCS	C	FMA-UE FMA-LE	1.32 ± 0.53 m	1.28 ± 0.58 m
41	Zhang Y (58)	2019	47.9 ± 6.7	48.5 ± 6.4	36	36	RRT + tDCS	C	FMA-UE	4.6 ± 1.5 m	4.3 ± 1.7 m
42	Wang W (59)	2021	60 ± 14	56 ± 11	15	15	PT + Tdcs	C	FMA-UE	20 ± 14d	20 ± 12d
43	Zhao F (60)	2021	60.4 ± 9.25	58.70 ± 9.64	39	39	VRT + tDCS	C	FMA-UE	38.3 ± 8.94d	34.7 ± 12.35d
44	Zhao JY (61)	2023	54.37 ± 4.54	54.98 ± 4.32	20	20	tDCS	C	FMA-UE FMA-LE	13.87 ± 2.42d	13.65 ± 2.31d
45	Zheng CJ (62)	2019	59.8 ± 8.3	61,1 ± 7.4	49	47	tDCS	C	FMA-UE	31.4 ± 11.4d	35.3 ± 12.2d
46	Zheng S (63)	2020	48.9 ± 2.0	48.5 ± 2.4	40	40	AM+tDCS	tDCS	FMA-UE	4.2 ± 2.9 m	4.6 ± 2.8 m
47	Zhou YP (64)	2018	54.25 ± 8.23	53.87 ± 8.91	32	31	MIT + Tdcs	C	FMA-UE	36.16 ± 19.9d	34.83 ± 22.91d
48	Alisar (65)	2020	63.56 ± 10.19	63.5 ± 12.6	16	16	tDCS	C	FMA-UE	352.62 ± 390.32d	442.75 ± 687.43d
49	Cha (14)	2014	59.8 ± 10.4	57.8 ± 9.9	10	10	tDCS	C	FMA-UE FMA-LE	13.8 ± 4.6 m	14.5 ± 3.6 m
50	Cho (66)	2015	58.29 ± 10.67	60.38 ± 10.19	14	13	MT + tDCS	C	FMA-UE	13.2 ± 5.1 m	15.5 ± 7.8 m
51	Zeng (67)	2024	55.14 ± 14.76	57.24 ± 13.75	21	21	tDCS	C	FMA-UE ARAT	1.25w	2w
52	Gong (68)	2023	56.3 ± 2.1	56.8 ± 2.8	37	35	tDCS	C	FMA-UE	49.6 ± 6.4d	48.3 ± 6.5d
53	Lee (69)	2014	63.1 ± 10.3	60.3 ± 11.3	20	19	VRT + tDCS	tDCS	FMA-UE	17.8 ± 7.3d	17.4 ± 9.4d
54	Tedla (15)	2022	58.5 ± 6.42	58.78 ± 5.46	18	18	PT + tDCS	C	FMA-UE	62.56 ± 17.1d	62.78 ± 20.98d
55	Hsu (70)	2023	59.1 ± 11.4	59.2 ± 11.8	13	14	tDCS	C	FMA-UE FMA-LE ARAT	20.7 ± 3.5d	21.1 ± 5.3d
56	Rabadi (71)	2020	62 ± 11	63 ± 6	8	8	tDCS	C	ARAT	6.9 ± 3.7 m	5.9 ± 2.8 m
57	Qurat (72)	2023	59 ± 4.61	57.95 ± 5.45	22	22	tDCS	C	BBS	16.5 ± 11.8 m	16.41 ± 10.26 m
58	Li (73)	2024	58.65 ± 12.677	56.54 ± 10.428	26	26	tDCS	C	FMA-UE ARAT	100.12d	87.58d
59	Lindenberg (13)	2010	61.7 ± 14.7	55.8 ± 12.9	10	10	tDCS	C	FMA-UE	30.5 ± 21.4 m	40.3 ± 23.4 m
60	Llorens (74)	2021	57.6 ± 6.9	52.3 ± 10.9	14	15	VRT + tDCS	C	FMA-UE	8.7 ± 2.3 m	9.3 ± 2.4 m
61	Chang (75)	2015	59.9 ± 10.2	65.8 ± 10.6	12	12	tDCS	C	FMA-LE BBS	16 ± 6.2d	16.6 ± 5.2d
62	Duan (76)	2023	65.83 ± 8.8	66.58 ± 10.3	46	45	tDCS	C	FMA-LE	5 ± 2.9w	4 ± 2.9w
63	Youssef (77)	2023	65	66	11	11	tDCS	C	FMA-UE FMA-LE BBS	NR	NR
64	Toktas (78)	2024	58.79 ± 10.19	62.57 ± 8.53	14	14	tDCS	C	FMA-LE BBS	8.07 ± 5.37 m	6.86 ± 3.08 m
65	Allman (79)	2016	59.5 ± 12.1	66.8 ± 10.4	11	13	tDCS	C	FMA-UE ARAT	51.2 ± 33.4 m	56.6 ± 39.8 m
66	Dinesh (80)	2011	61 ± 12	56 ± 15	7	7	tDCS	C	FMA-UE	33 ± 20 m	28 ± 28 m
67	Fusco (81)	2014	56.4	60	5	6	tDCS	C	FMA-UE	NR	NR
68	Kim (82)	2010	55.3 ± 16.4	62.9 ± 9.2	6	7	tDCS	C	FMA-UE	34 ± 27.1d	22.9 ± 7.5d
69	Oveisgharan (83)	2017	52.1 ± 12.8	65.3 ± 16.5	10	10	tDCS	C	FMA-UE ARAT	2.1 ± 3d	3.8 ± 5.8d
70	Pinto (84)	2021	45.6 ± 12.1	48.1 ± 9.4	31	29	tDCS	C	FMA-UE FMA-LE	1-411d	1-411d
71	Prathum (85)	2022	58.67 ± 3.7	56.83 ± 3.58	12	12	tDCS	C	FMA-UE FMA-LE	15.5 ± 2.6 m	16.33 ± 3.3 m
72	Rossi (86)	2013	66.1 ± 14.3	70.3 ± 13.5	25	25	tDCS	C	FMA-UE	9.8 ± 2.4d	9.5 ± 2.8d
73	Lazzaro (87)	2014	71.71 ± 5.25	66.43 ± 5.96	7	7	tDCS	C	ARAT	2.71 ± 0.42d	2.57 ± 0.81d
74	Kim (88)	2024	65.78 ± 12.6	57.13 ± 9.49	9	8	tDCS	C	BBS	NR	NR

TG, treatment group; CG, control group; FMA-UE, Fugl-Meyer Assessment for Upper Extremity; ARAT, Action Research Arm Test; FMA-LE, Fugl-Meyer Assessment for Lower Extremity; BBS, Berg Balance Scale. tDCS, transcranial direct current stimulation; C, conventional rehabilitation; MIT, motor imagery therapy; FES, functional electrical stimulation; MT, mirror therapy; AM, acupuncture and moxibustion; CIMT, constraint-induced movement therapy; BCIT, brain-computer interface therapy; VRT, virtual reality technology; RRT, robotic rehabilitation therapy; PT, proprioceptive training. NR, no report.

### 3.3 Quality assessment of the included studies

**Figure 2:**
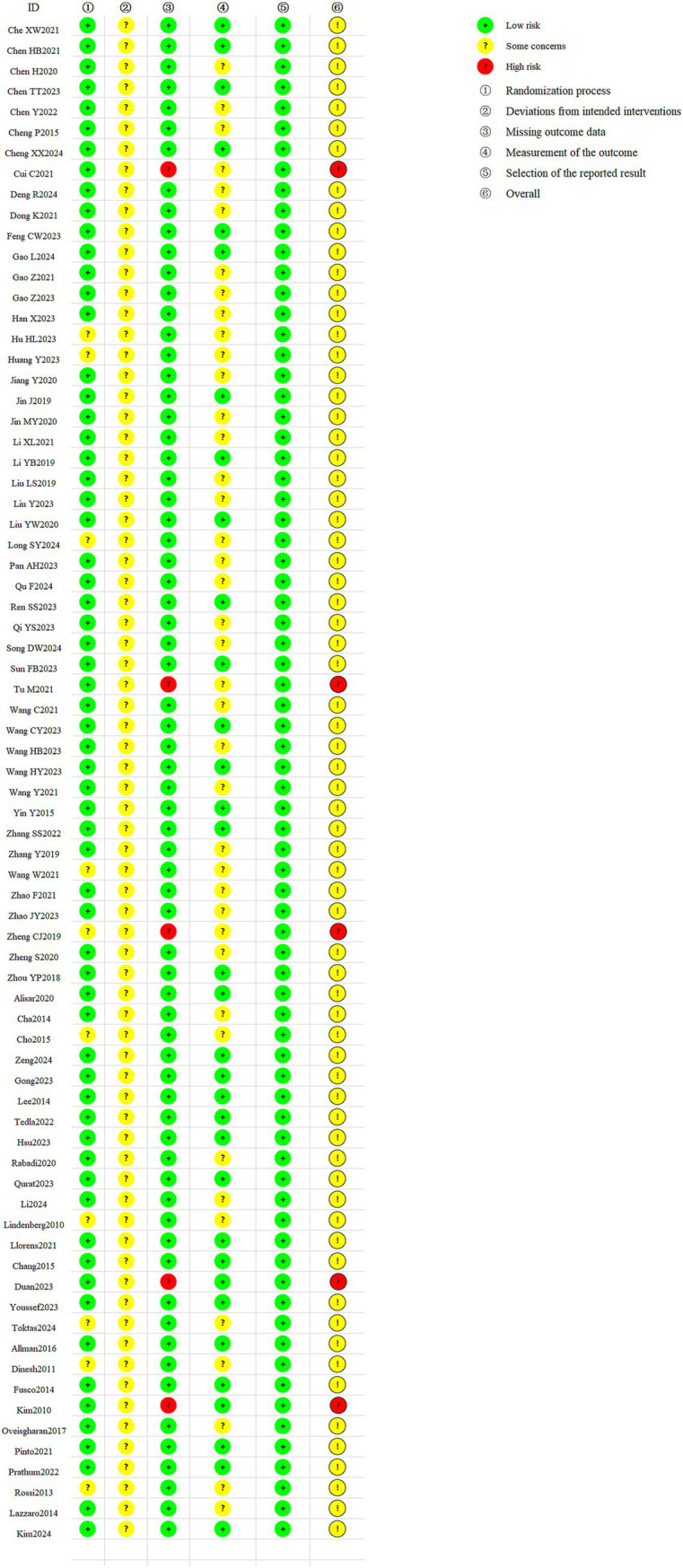
Quality assessment of included studies.

Regarding the overall risk of bias, 69 studies (13–15, 19–49, 51–61, 63–75, 77–81, 83–88) were assessed with moderate risk of bias, while 5 studies (16, 50, 62, 76, 82) were found to have high risk of bias. In terms of randomization, 64 studies had low risk of bias, and 10 studies had moderate risk. All studies showed moderate risk of bias concerning whether the intervention deviated from the intended plan. Regarding missing outcome data, 69 studies had low risk of bias, and 5 had high risk. For outcome measurement, 33 studies had low risk of bias, and 41 had moderate risk. Regarding selective outcome reporting, all studies exhibited low risk of bias. In conclusion, the main sources of bias in the included studies were moderate bias due to deviations from the intended interventions and high bias resulting from missing outcome data. A detailed description of the risk of bias for the included studies is provided in Figure 2.

### 3.4 Network meta-analysis

**Figure 3:**
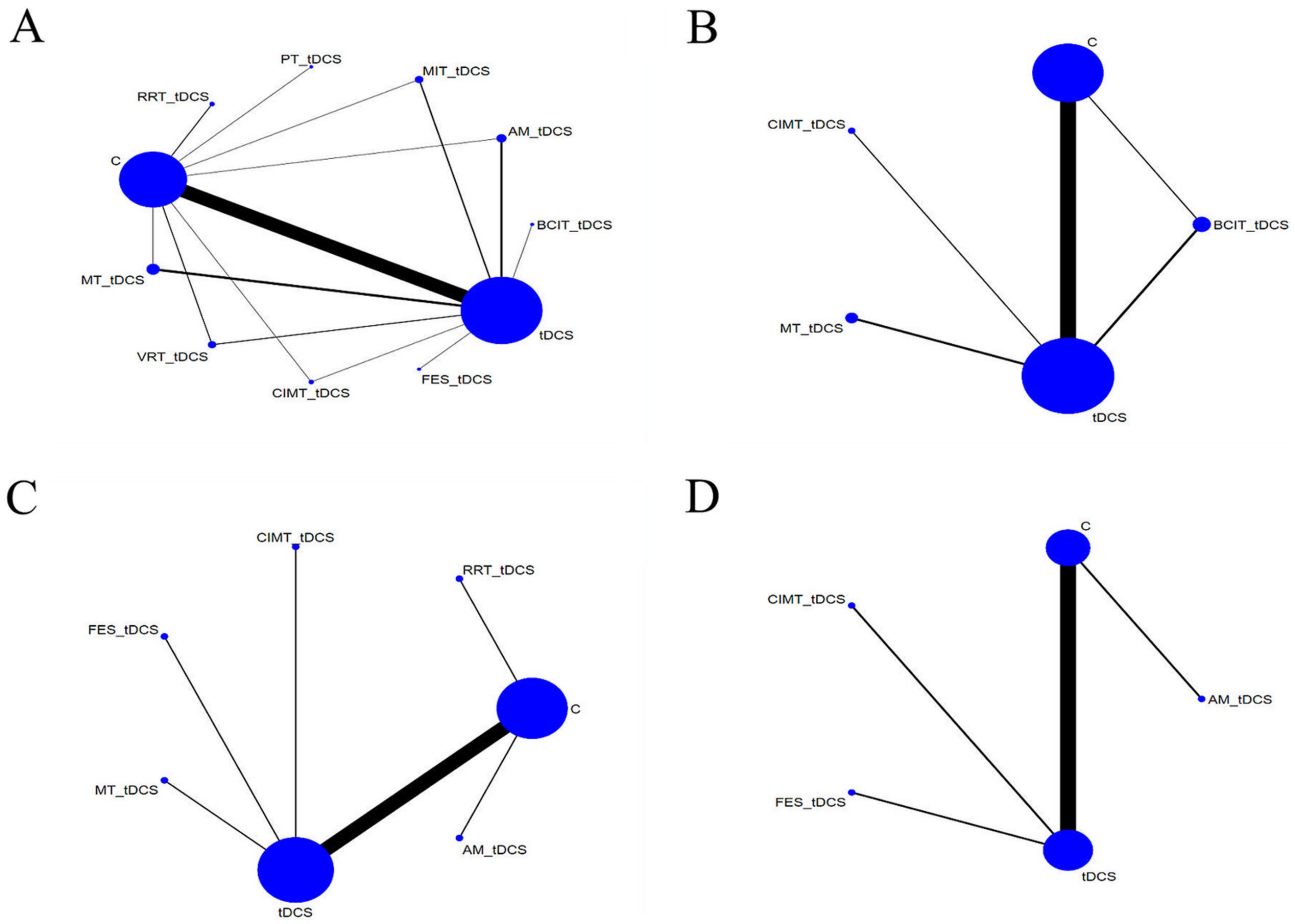
Network evidence diagrams **(A)** FMA-UE; **(B)** ARAT; **(C)** FMA-LE; **(D)** BBS. FMA-UE, Fugl-Meyer Assessment for Upper Extremity; ARAT, Action Research Arm Test; FMA-LE, Fugl-Meyer Assessment for Lower Extremity; BBS, Berg Balance Scale. tDCS, transcranial direct current stimulation; C, conventional rehabilitation; MIT, motor imagery therapy; FES, functional electrical stimulation; MT, mirror therapy; AM, acupuncture and moxibustion; CIMT, constraint-induced movement therapy; BCIT, brain-computer interface therapy; VRT, virtual reality technology; RRT, robotic rehabilitation therapy; PT, proprioceptive training.

Figure 3 presents the network evidence diagrams. In the network evidence diagram, each node represents a different intervention, with the size of the node indicating the sample size of that intervention. The connections between nodes reflect the number of studies comparing the corresponding interventions, with line thickness increasing as the study count rises.

#### 3.4.1 Fugl-Meyer assessment-upper extremity

A total of 60 studies (13–15, 19, 21–26, 28–36, 38–47, 49–51, 54–70, 73, 74, 77, 79–86) reported Fugl-Meyer Assessment-Upper Extremity (FMA-UE) scores, covering 11 rehabilitation strategies, including RRT + tDCS, PT + tDCS, MIT + tDCS, AM+tDCS, BCIT+tDCS, tDCS, FES + tDCS, CIMT+tDCS, VRT + tDCS, MT + tDCS, and C. The network evidence diagram for FMA-UE is illustrated in Figure 3A, while the corresponding league table is provided in Table 2. Regarding the enhancement of FMA-UE scores, the following interventions showed better therapeutic effects compared to C: AM+tDCS (MD: 10.72, 95% CrI: 6.96–14.75), BCIT+tDCS (MD: 17.76, 95% CrI: 6.63–28.87), CIMT+tDCS (MD: 9.04, 95% CrI: 3.66–14.45), FES + tDCS (MD: 14.7, 95% CrI: 7.08–22.45), MIT + tDCS (MD: 13.2, 95% CrI: 8.86–17.63), MT + tDCS (MD: 13.08, 95% CrI: 9.79–16.36), PT + tDCS (MD: 8.21, 95% CrI: 0.84–15.55), RRT + tDCS (MD: 7.31, 95% CrI: 1.79–12.94), tDCS (MD: 5.67, 95% CrI: 4.22–7.19) and VRT + tDCS (MD: 8.49, 95% CrI: 4.57–12.4). Moreover, compared to tDCS alone, the following interventions demonstrated better effects: AM+tDCS (MD: 5.05, 95% CrI: 1.42–8.86), BCIT+tDCS (MD: 12.09, 95% CrI: 1.05–23.08), FES + tDCS (MD: 9.04, 95% CrI: 1.49–16.6), MIT + tDCS (MD: 7.53, 95% CrI: 3.32–11.74) and MT + tDCS (MD: 7.42, 95% CrI: 4.34–10.35). All differences were statistically significant.

**Table 2:** League table of FMA-UE and ARAT.

MD 95% CrI												
ARAT	AM+tDCS	7 (−4.72, 18.57)	−10.72 (−14.75, −6.96)	−1.68 (−8.26, 4.71)	4 (−4.54, 12.29)	2.47 (−3.19, 8.02)	2.35 (−2.55, 7.01)	−2.51 (−10.98, 5.67)	−3.41 (−10.26, 3.32)	−5.05 (−8.86, −1.42)	−2.23 (−7.72, 3.01)	FMA-UE
		BCIT+tDCS	−17.76 (−28.87, −6.63)	−8.7 (−20.95, 3.54)	−3.04 (−16.37, 10.35)	−4.54 (−16.29, 7.24)	−4.68 (−16.13, 6.74)	−9.53 (−22.88, 3.77)	−10.45 (−22.81, 2.09)	−12.09 (−23.08, −1.05)	−9.27 (−20.99, 2.4)	
		2.64 (−6.58, 12.37)	C	9.04 (3.66, 14.45)	14.7 (7.08, 22.45)	13.2 (8.86, 17.63)	13.08 (9.79, 16.36)	8.21 (0.84, 15.55)	7.31 (1.79, 12.94)	5.67 (4.22, 7.19)	8.49 (4.57, 12.4)	
		−4.78 (−19.83, 10.97)	−7.38 (−20.49, 5.66)	CIMT+tDCS	5.69 (−3.58, 14.94)	4.15 (−2.64, 10.99)	4.04 (−2.17, 10.14)	−0.83 (−9.93, 8.25)	−1.75 (−9.43, 6.05)	−3.38 (−8.75, 2.03)	−0.56 (−7.16, 6.02)	
					FES + tDCS	−1.53 (−10.19, 7.14)	−1.64 (−9.82, 6.41)	−6.5 (−17.18, 4.06)	−7.41 (−16.88, 2.09)	−9.04 (−16.6, −1.49)	−6.23 (−14.77, 2.25)	
						MIT + tDCS	−0.12 (−5.35, 4.99)	−4.97 (−13.62, 3.53)	−5.89 (−12.97, 1.23)	−7.53 (−11.74, −3.32)	−4.71 (−10.53, 0.96)	
		−7.81 (−20.37, 5.48)	−10.44 (−20.41, −0.25)	−3.04 (−18.24, 12.54)			MT + tDCS	−4.88 (−12.86, 3.17)	−5.78 (−12.14, 0.82)	−7.42 (−10.35, −4.34)	−4.59 (−9.49, 0.33)	
								PT + tDCS	−0.9 (−10.07, 8.41)	−2.54 (−9.98, 5)	0.28 (−8.05, 8.58)	
									RRT + tDCS	−1.64 (−7.43, 4.11)	1.19 (−5.72, 7.91)	
		−1.95 (−10.8, 7.36)	−4.6 (−8.82, −0.42)	2.81 (−9.67, 15.16)			5.84 (−3.48, 14.86)			tDCS	2.82 (−1.14, 6.68)	
											VRT + tDCS	

FMA-UE, Fugl-Meyer Assessment for Upper Extremity; ARAT, Action Research Arm Test; tDCS, transcranial direct current stimulation; C, conventional rehabilitation; MIT, motor imagery therapy; FES, functional electrical stimulation; MT, mirror therapy; AM, acupuncture and moxibustion; CIMT, constraint-induced movement therapy; BCIT, brain-computer interface therapy; VRT, virtual reality technology; RRT, robotic rehabilitation therapy; PT, proprioceptive training.

#### 3.4.2 Action research arm test

A total of 16 studies (29, 36, 38–40, 44, 48, 52, 56, 67, 70, 71, 73, 79, 83, 87) reported Action Research Arm Test (ARAT) scores, involving five rehabilitation strategies: C, BCTT+tDCS, tDCS, MT + tDCS, and CIMT+tDCS. The network evidence diagram for ARAT is illustrated in Figure 3B, while the corresponding league table is provided in Table 2. Regarding the enhancement of ARAT scores, the following interventions showed better therapeutic effects compared to C: MT + tDCS (MD: 10.44, 95% CrI: 0.25–20.41) and tDCS (MD: 4.6, 95% CrI: 0.42–8.82). All differences were statistically significant.

#### 3.4.3 Fugl-Meyer assessment-lower extremity

A total of 16 studies (14, 20, 23, 37, 43, 45, 53, 57, 61, 70, 75–78, 84, 85) reported Fugl-Meyer Assessment-Lower Extremity (FMA-LE) scores, involving seven rehabilitation strategies: MT + tDCS, FES + tDCS, CIMT+tDCS, RRT + tDCS, C, AM+tDCS, and tDCS. The network evidence diagram for FMA-LE is illustrated in Figure 3C, while the corresponding league table is provided in Table 3. Regarding the enhancement of FMA-LE scores, the following interventions showed better therapeutic effects compared to C: AM+tDCS (MD: 6.7, 95% CrI: 0.63–12.8), MT + tDCS (MD: 7.82, 95% CrI: 1.19–14.57) and tDCS (MD: 2.52, 95% CrI: 0.64–4.49). All differences were statistically significant.

**Table 3:** League table of FMA-LE and BBS.

MD 95% CrI								
BBS	AM + tDCS	−6.7 (−12.8, −0.63)	−2.5 (−11.12, 6.28)	−2.66 (−12.34, 7.1)	1.11 (−7.87, 10.17)	−4.19 (−12.75, 4.31)	−4.18 (−10.51, 2.23)	FMA-LE
	8.22 (−2.2, 18.63)	C	4.21 (−1.96, 10.44)	4.05 (−3.45, 11.7)	7.82 (1.19, 14.57)	2.51 (−3.45, 8.47)	2.52 (0.64, 4.49)	
	2.13 (−13.47, 17.58)	−6.07 (−17.86, 5.44)	CIMT + tDCS	−0.15 (−9.54, 9.25)	3.61 (−5.11, 12.29)	−1.69 (−10.37, 6.87)	−1.68 (−7.58, 4.2)	
	2.97 (−13.18, 18.88)	−5.26 (−17.6, 6.96)	0.83 (−15.1, 16.71)	FES + tDCS	3.75 (−5.98, 13.49)	−1.55 (−11.23, 8.04)	−1.53 (−8.87, 5.79)	
					MT + tDCS	−5.3 (−14.31, 3.63)	−5.29 (−11.72, 1.09)	
						RRT + tDCS	0.01 (−6.21, 6.31)	
	4.58 (−6.72, 15.69)	−3.64 (−7.88, 0.39)	2.42 (−8.4, 13.29)	1.6 (−10.02, 13.18)			tDCS	

FMA-LE, Fugl-Meyer Assessment for Lower Extremity; BBS, Berg Balance Scale; tDCS, transcranial direct current stimulation; C, conventional rehabilitation; AM, acupuncture and moxibustion; CIMT, constraint-induced movement therapy; FES, functional electrical stimulation; MT, mirror therapy; RRT, robotic rehabilitation therapy.

#### 3.4.4 Berg balance scale

A total of 10 studies (16, 20, 27, 37, 53, 72, 75, 77, 78, 88) reported Berg Balance Scale (BBS) scores, involving four rehabilitation strategies: CIMT+tDCS, FES + tDCS, C, tDCS, and AM+tDCS. The network evidence diagram for BBS is illustrated in Figure 3D, while the corresponding league table is provided in Table 3. Regarding the enhancement of BBS scores, none of the rehabilitation strategies demonstrated statistically significant differences in effectiveness.

### 3.5 SUCRA ranking

**Figure 4:**
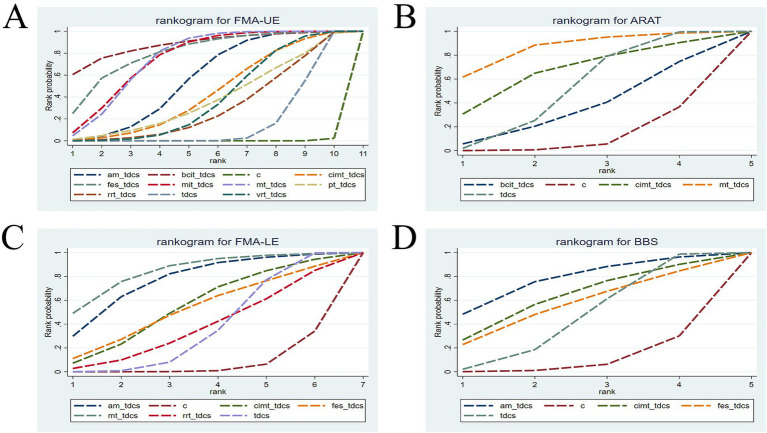
Cumulative probability ranking charts **(A)** FMA-UE; **(B)** ARAT; **(C)** FMA-LE; **(D)** BBS. FMA-UE, Fugl-Meyer Assessment for Upper Extremity; ARAT, Action Research Arm Test; FMA-LE, Fugl-Meyer Assessment for Lower Extremity; BBS, Berg Balance Scale. tDCS, transcranial direct current stimulation; C, conventional rehabilitation; MIT, motor imagery therapy; FES, functional electrical stimulation; MT, mirror therapy; AM, acupuncture and moxibustion; CIMT, constraint-induced movement therapy; BCIT, brain-computer interface therapy; VRT, virtual reality technology; RRT, robotic rehabilitation therapy; PT, proprioceptive training.

The cumulative probability rankings for the four outcome measures are presented in Figure 4 and Table 4. The results indicate that BCIT+tDCS (SUCRA = 88.34%) is the most effective tDCS-based combined intervention for improving FMA-UE scores, followed by FES + tDCS (SUCRA = 80.99%), MT + tDCS (SUCRA = 75.83%), MIT + tDCS (SUCRA = 75.80%), AM+tDCS (SUCRA = 57.18%), CIMT+tDCS (SUCRA = 44.11%), VRT + tDCS (SUCRA = 39.31%), PT + tDCS (SUCRA = 39.07%), RRT + tDCS (SUCRA = 31.75%), tDCS (SUCRA = 17.39%), and C (SUCRA = 0.23%). Regarding ARAT scores, MT + tDCS (SUCRA = 85.96%) demonstrated the best efficacy, ranking above CIMT+tDCS (SUCRA = 66.38%), tDCS (SUCRA = 51.51%), BCIT+tDCS (SUCRA = 35.38%), and C (SUCRA = 10.76%). For FMA-LE scores, MT + tDCS (SUCRA = 84.29%) was identified as the most effective intervention, followed by AM+tDCS (SUCRA = 76.94%), CIMT+tDCS (SUCRA = 55.02%), FES + tDCS (SUCRA = 52.45%), RRT + tDCS (SUCRA = 37.57%), tDCS (SUCRA = 36.79%), and C (SUCRA = 6.94%). In terms of BBS scores, AM+tDCS (SUCRA = 77.16%) ranked highest, followed by CIMT+tDCS (SUCRA = 62.47%), FES + tDCS (SUCRA = 55.79%), tDCS (SUCRA = 45.19%), and C (SUCRA = 9.39%).

**Table 4:** Cumulative probability ranking table.

Intervention	FMA-UE	ARAT	FMA-LE	BBS
AM + tDCS	57.18	0	76.94	77.16
BCIT + tDCS	88.34	35.38	0	0
C	0.23	10.76	6.94	9.39
CIMT + tDCS	44.11	66.38	55.02	62.47
FES + tDCS	80.99	0	52.45	55.79
MIT + tDCS	75.80	0	0	0
MT + tDCS	75.83	85.96	84.29	0
PT + tDCS	39.07	0	0	0
RRT + tDCS	31.75	0	37.57	0
tDCS	17.39	51.51	36.79	45.19
VRT + tDCS	39.31	0	0	0

FMA-UE, Fugl-Meyer Assessment for Upper Extremity; ARAT, Action Research Arm Test; FMA-LE, Fugl-Meyer Assessment for Lower Extremity; BBS, Berg Balance Scale. tDCS, transcranial direct current stimulation; C, conventional rehabilitation; MIT, motor imagery therapy; FES, functional electrical stimulation; MT, mirror therapy; AM, acupuncture and moxibustion; CIMT, constraint-induced movement therapy; BCIT, brain-computer interface therapy; VRT, virtual reality technology; RRT, robotic rehabilitation therapy; PT, proprioceptive training.

### 3.6 Two-dimensional clustering analysis

**Figure 5:**
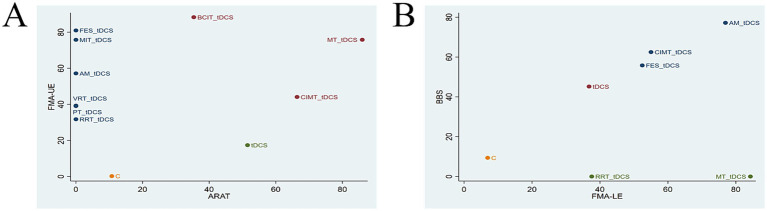
Clustering analysis charts **(A)** FMA-UE and ARAT; **(B)** FMA-LE and BBS. FMA-UE, Fugl-Meyer Assessment for Upper Extremity; ARAT, Action Research Arm Test; FMA-LE, Fugl-Meyer Assessment for Lower Extremity; BBS, Berg Balance Scale. tDCS, transcranial direct current stimulation; C, conventional rehabilitation; MIT, motor imagery therapy; FES, functional electrical stimulation; MT, mirror therapy; AM, acupuncture and moxibustion; CIMT, constraint-induced movement therapy; BCIT, brain-computer interface therapy; VRT, virtual reality technology; RRT, robotic rehabilitation therapy; PT, proprioceptive training.

The two-dimensional clustering analysis for the four outcome measures is shown in Figure 5. As depicted in Figure 5A, MT + tDCS (SUCRA = 75.83%/85.96%) demonstrated significant efficacy in improving both FMA-UE and ARAT scores. FMA-UE evaluates upper limb motor ability in PSSS, while ARAT assesses arm mobility. Together, these two scales comprehensively reflect upper limb function in PSSS. Figure 5B indicates that AM+tDCS (SUCRA = 76.94%/77.16%), FES + tDCS (SUCRA = 52.45%/55.79%), and CIMT+tDCS (SUCRA = 55.02%/62.47%) exhibited notable effectiveness in enhancing FMA-LE and BBS scores. FMA-LE evaluates lower limb motor ability in PSSS, while BBS assesses lower limb balance. Together, these two scales provide a comprehensive reflection of lower limb function in PSSS. In summary, MT + tDCS (SUCRA = 75.83%/85.96%) may represent the optimal intervention for upper limb dysfunction in PSSS, while AM+tDCS (SUCRA = 76.94%/77.16%) may be the most effective strategy for improving lower limb function in PSSS.

### 3.7 Publication bias

**Figure 6:**
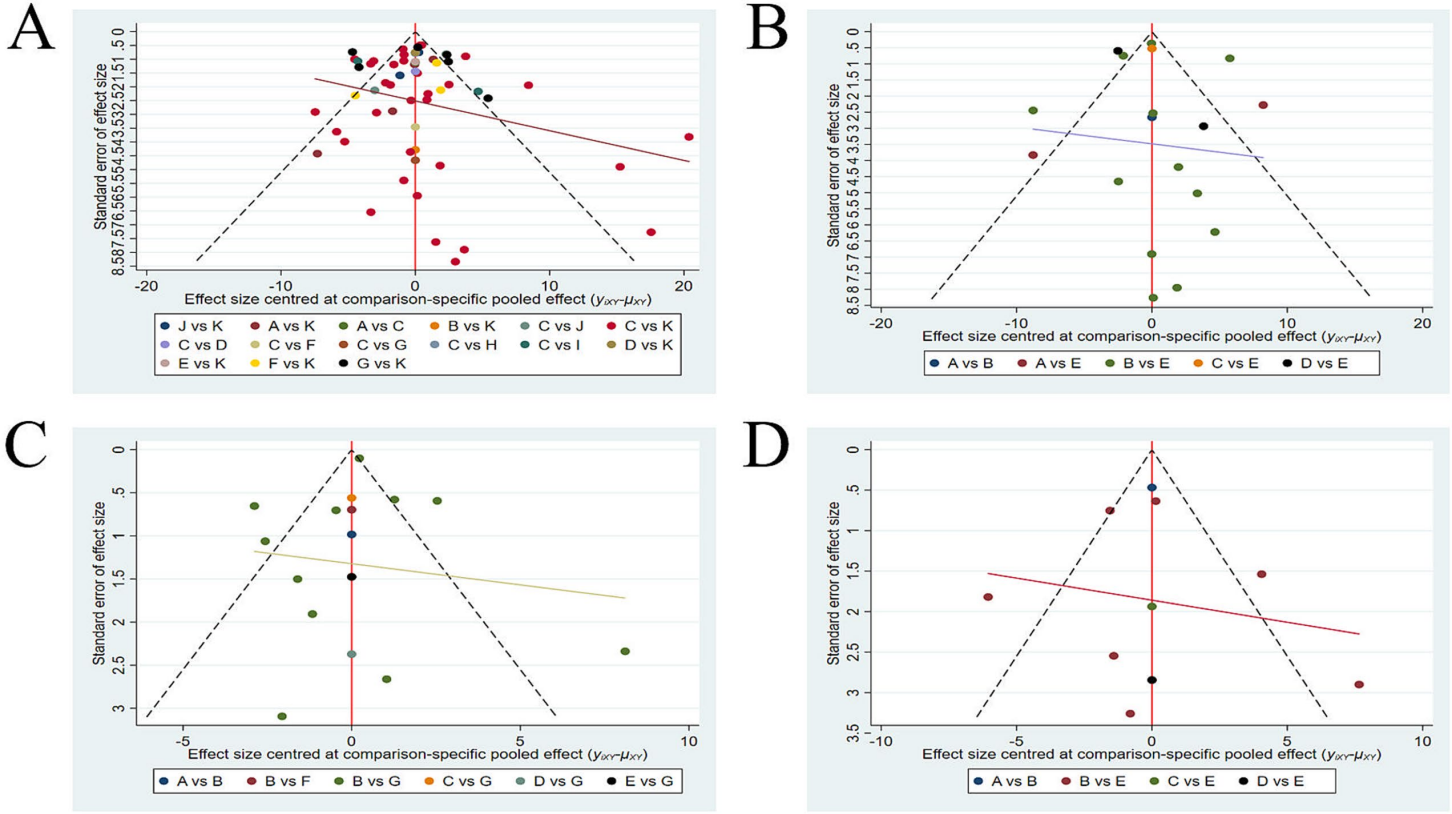
Funnel plots **(A)** FMA-UE; **(B)** ARAT; **(C)** FMA-LE; **(D)** BBS. FMA-UE, Fugl-Meyer Assessment for Upper Extremity; ARAT, Action Research Arm Test; FMA-LE, Fugl-Meyer Assessment for Lower Extremity; BBS, Berg Balance Scale.

The funnel plots for the four outcome measures are shown in Figure 6. In Figures 6A,C,D, the plots appear symmetrical overall, with only a few data points falling outside the funnel. It suggests a low likelihood of publication bias for the FMA-UE, FMA-LE, and BBS outcomes. However, in Figure 6B, a lot of data points fall outside the funnel, indicating a higher probability of publication bias for the ARAT outcome. To further assess whether the publication bias in ARAT outcomes was significant, we conducted Begg’s test. The result of Begg’s test showed that *p* = 0.910 (*p* > 0.05), indicating a low likelihood of publication bias for the ARAT outcome.

### 3.8 Regression analysis

As shown in Table 5, the results of the regression analysis between intervention effects and publication year indicate that the credible interval for all four outcome measures do not cross zero. This suggests that publication year is not a moderating factor influencing the treatment effects of the intervention strategies.

**Table 5:** Regression analysis between publication year and intervention effect.

Outcomes	95% CrI
FMA-UE	(−2.5145, 3.213)
ARAT	(−9.498, 6.0205)
FMA-LE	(−1.0525, 5.551)
BBS	(−2.6679, 9.772)

FMA-UE, Fugl-Meyer Assessment for Upper Extremity; ARAT, Action Research Arm Test; FMA-LE, Fugl-Meyer Assessment for Lower Extremity; BBS, Berg Balance Scale.

### 3.9 Convergence diagnostics

**Figure 7:**
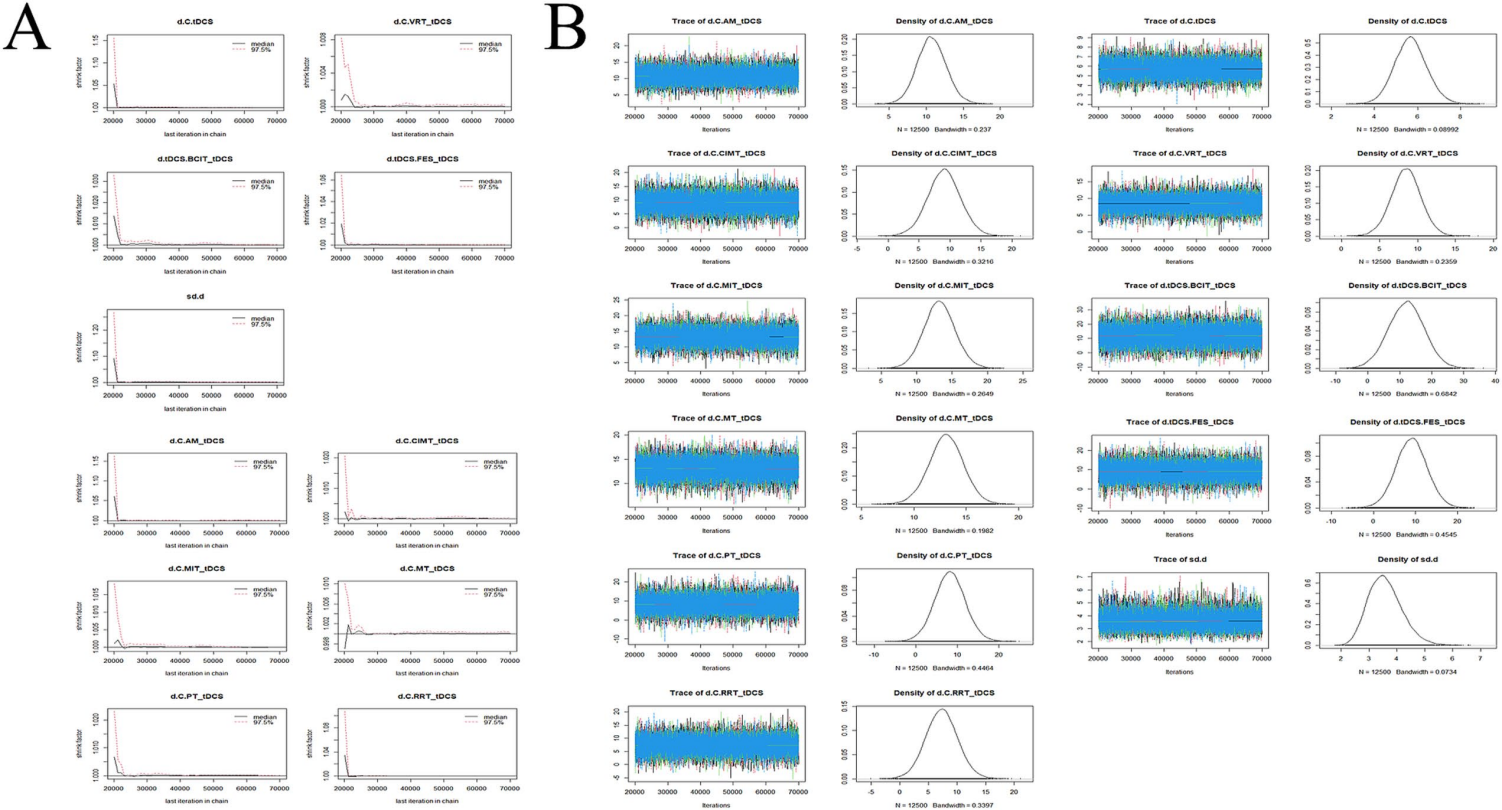
Convergence diagnostic plots, trace plots, and density plots of FMA-UE **(A)** convergence diagnostic plots; **(B)** trace plots and density plots. FMA-UE, Fugl-Meyer Assessment for Upper Extremity; C, conventional rehabilitation; tDCS, transcranial direct current stimulation; MIT, motor imagery therapy; FES, functional electrical stimulation; MT, mirror therapy; AM, acupuncture and moxibustion; CIMT, constraint-induced movement therapy; BCIT, brain-computer interface therapy; VRT, virtual reality technology; RRT, robotic rehabilitation therapy; PT, proprioceptive training.

**Figure 8:**
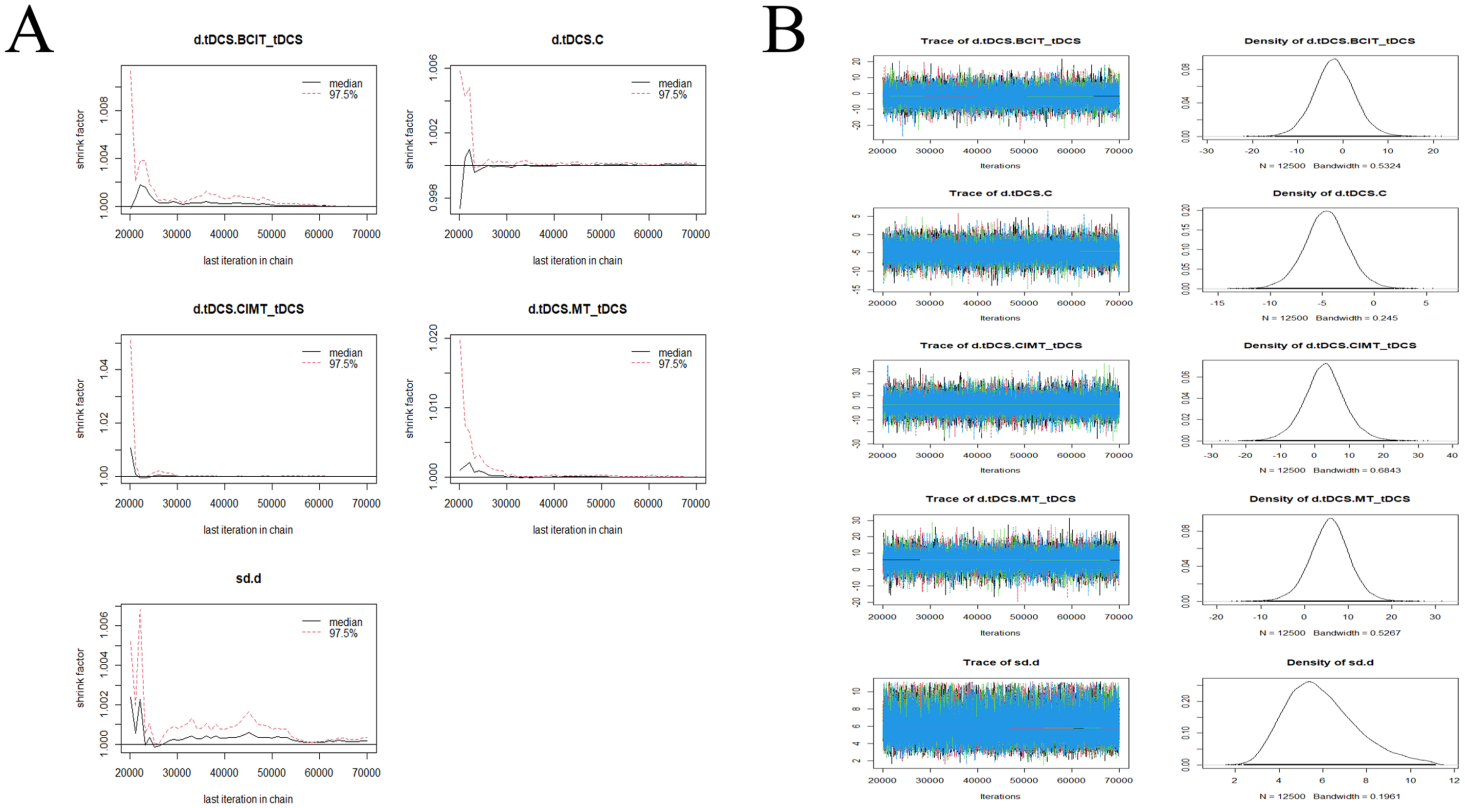
Convergence diagnostic plots, trace plots, and density plots of ARAT **(A)** convergence diagnostic plots; **(B)** trace plots and density plots. ARAT, Action Research Arm Test; C, conventional rehabilitation; tDCS, transcranial direct current stimulation; CIMT, constraint-induced movement therapy; BCIT, brain-computer interface therapy; MT, mirror therapy.

**Figure 9:**
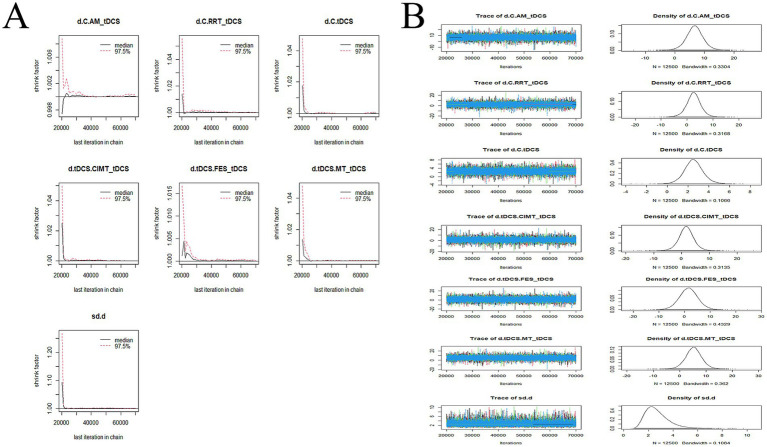
Convergence diagnostic plots, trace plots, and density plots of FMA-LE **(A)** convergence diagnostic plots; **(B)** trace plots and density plots. FMA-LE, Fugl-Meyer Assessment for Lower Extremity; C, conventional rehabilitation; tDCS, transcranial direct current stimulation; AM, acupuncture and moxibustion; RRT, robotic rehabilitation therapy; CIMT, constraint-induced movement therapy; FES, functional electrical stimulation; MT, mirror therapy.

**Figure 10:**
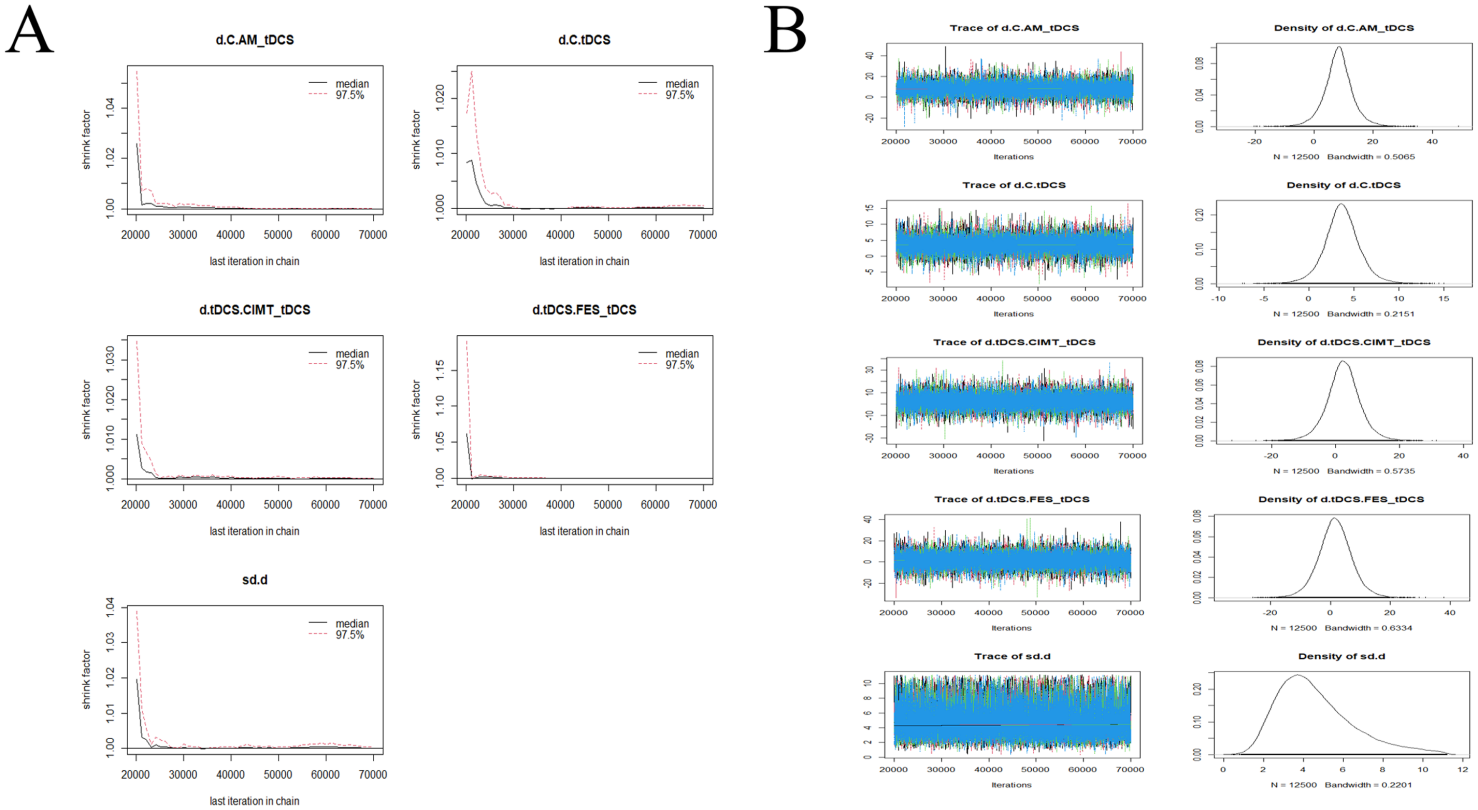
Convergence diagnostic plots, trace plots, and density plots of BBS **(A)** convergence diagnostic plots; **(B)** trace plots and density plots. BBS, Berg Balance Scale; C, conventional rehabilitation; tDCS, transcranial direct current stimulation; AM, acupuncture and moxibustion; CIMT, constraint-induced movement therapy; FES, functional electrical stimulation.

The convergence diagnostics and the trace and density plots are presented in Figures 7–10. We evaluated the convergence and stability of the model by drawing convergence diagnostic maps and trajectory density maps. Before 70,000 iterations, the convergence diagnostic plots and trace plots had converged, and the potential scale reduced factor tended to approach 1. The density plots was a smooth curve with a normal distribution, and the Bandwidth value approached 0, indicating satisfactory convergence and good stability of the model.

## 4 Discussion

This network meta-analysis (NMA) incorporated 74 randomized controlled trials (RCTs) with a total of 4,335 people suffering from stroke-related symptoms (PSSS) and nine tDCS-based combined intervention strategies. The network meta-analysis (NMA) results indicated that brain-computer interface therapy (BCIT) + tDCS (SUCRA = 88.34%) was the best intervention for enhancing upper limb motor ability in PSSS. For arm mobility and lower limb motor ability, mirror therapy (MT) + tDCS (SUCRA = 85.96%/84.29%) demonstrated the best efficacy, while acupuncture and moxibustion (AM) + tDCS (SUCRA = 77.16%) was the most effective for enhancing balance ability. The two-dimensional clustering analysis showed that MT + tDCS (SUCRA = 75.83%/85.96%) was the optimal tDCS-based rehabilitation strategy for treating upper limb motor dysfunction in PSSS, while AM+tDCS (SUCRA = 76.94%/77.16%) was the best tDCS-based rehabilitation strategy for improving lower limb motor dysfunction in PSSS.

BCIT+tDCS was identified as the most effective intervention for improving upper limb motor ability in PSSS. BCIT is an advanced communication system that integrates hardware and software, enabling the extraction, decoding, and translation of brain motor intention signals into commands that control external devices, thus facilitating direct brain-environment interaction (89, 90). BCIT has been widely used in the rehabilitation of upper limb motor ability in PSSS. Wang et al. (91) reported that BCIT could further enhance upper limb motor function in PSSS when combined with conventional rehabilitation therapy, compared to conventional rehabilitation alone. Additionally, meta-analyses conducted by Nojima et al. (92) and Li et al. (93) confirmed the superior efficacy of BCIT-based training over traditional rehabilitation in improving upper limb function in PSSS patients. In recent years, significant progress has been made in BCIT-based rehabilitation for motor function recovery in PSSS, and integrating BCIT with other rehabilitation strategies appears to be a promising and innovative approach. For instance, BCIT combined with robotic rehabilitation therapy (94) and BCIT combined with functional electrical stimulation therapy (95) have shown enhanced therapeutic effects. The development of these BCIT-based combination therapies supports the findings of this study, which identified BCIT+tDCS as the most effective strategy for improving upper limb motor ability in PSSS. Anodal tDCS applied before BCIT may pre-activate the ipsilesional hemisphere and excite the ipsilesional motor cortex, thereby facilitating faster BCIT activation and enhancing characteristic brain signals (96). Furthermore, tDCS creates a favorable neural environment, which may promote motor learning and enhance BCIT-induced motor learning capacity (97). BCIT, incorporating visual, auditory, and motor feedback, continuously reinforces motor imagery within the brain, providing peripheral visual, auditory, and proprioceptive feedback to the central nervous system. This process strengthens positive feedback loops, reinforces correct motor patterns, and promotes the reconstruction of damaged neural pathways, leading to enhanced therapeutic outcomes beyond those achieved with tDCS alone (98). Moreover, both BCIT and tDCS directly stimulate the central nervous system, enhancing neuroplasticity. Their combined application may produce a synergistic effect, maximizing cortical excitability and neuroplasticity, which could further improve upper limb motor ability in PSSS.

MT + tDCS is considered one of the effective combined treatment approaches for improving arm mobility and lower limb motor ability in PSSS patients. Mirror Therapy (MT), as a neurorehabilitation technique, uses visual illusions created by mirror reflection, imitation, and motor imagery, allowing the brain to perceive the movement of the affected limb as normal (99, 100). Although this process theoretically may involve the activation of the mirror neuron system, thereby facilitating functional recovery of the affected side, it should be noted that the RCTs included in this study did not provide direct neuroimaging or electrophysiological data to support this mechanism. Therefore, we describe this mechanism as a potential explanation. In fact, the multisensory stimulation provided by MT not only potentially activates the mirror neuron system (101), but also enhances the excitability of the primary motor cortex (M1) (102), promotes cortical reorganization, and improves motor cortex plasticity through repeated high-intensity visual feedback, thereby mitigating the phenomenon of “learned non-use” of the affected limb. Furthermore, MT emphasizes bilateral symmetrical movements, which helps to more widely activate the motor cortex, facilitating the activation of residual motor pathways on the affected side (103, 104), thereby promoting motor recovery. In PSSS, an interhemispheric imbalance is often observed, wherein the affected hemisphere exhibits reduced excitability, while the unaffected hemisphere exerts excessive inhibition via the transcallosal pathway (105). Anodal tDCS enhances the excitability of the affected M1 and corrects the pathological interhemispheric inhibition, which may contribute to the restoration of lower limb motor ability and arm mobility. Considering that patients with PSSS often exhibit reduced excitability in the affected hemisphere and excessive inhibition from the healthy hemisphere, anodal tDCS may help correct this interhemispheric imbalance by enhancing the excitability of the affected M1, thus playing a positive role in improving both arm mobility and lower limb motor function (21). Applying anodal tDCS prior to MT may pre-activate the relevant brain regions, creating a more favorable neural environment for subsequent mirror neuron activation, thereby enhancing the efficiency of neural network reorganization (21).

AM combined with tDCS has been recognized as an effective combined therapeutic strategy for improving balance function in PSSS. Acupuncture and moxibustion (AM) is a therapeutic approach based on traditional Chinese medicine principles, involving the insertion of needles at specific angles into the body and applying techniques such as twisting and lifting to stimulate targeted areas for therapeutic effects. AM can enhance excitability in brain regions affected by pathological changes, promote the establishment of collateral cerebral circulation, rapidly alleviate vascular spasms, induce vasodilation, reduce vascular resistance, and increase cerebral blood flow. These effects contribute to alleviating ischemia in brain tissues surrounding the lesion, rescuing ischemic but functionally impaired neurons, and accelerating central nervous system repair and reconstruction (106). However, the proposed mechanisms underlying AM are primarily based on traditional theories and preliminary literature, lacking direct evidence from modern neuroimaging studies. Therefore, these mechanisms remain hypothetical and warrant further investigation through contemporary mechanistic research. The combination of tDCS and AM may further enhance neuroplasticity and functional recovery through synergistic effects. tDCS modulates cortical excitability, increasing activity in the damaged motor cortex while inhibiting excessive excitability in the unaffected hemisphere, thereby restoring interhemispheric balance. Meanwhile, AM stimulates specific acupoints, activating intracerebral neural networks, improving local blood circulation, and facilitating the release of neurotrophic factors, collectively promoting central nervous system repair (57). Additionally, AM reduces pain, alleviates muscle spasticity, and promotes muscle strength recovery in the affected limbs, which further contributes to improved balance ability in PSSS (54). It is noteworthy that all studies involving AM+tDCS were conducted in China, and there may be considerable variation in the specific acupuncture procedures employed—such as acupoint selection, stimulation intensity, needle angle, and frequency of manipulation. These differences could influence the therapeutic outcomes and limit the generalizability of this combined intervention across diverse cultural or clinical settings. Future cross-cultural and multicenter studies are warranted, not only to investigate the relationship between acupuncture parameters and brain functional remodeling using modern neuroimaging techniques, but also to explore the acceptability and efficacy of different acupuncture modalities in varied cultural contexts. Such efforts would provide a more comprehensive foundation for the broader clinical application of AM+tDCS.

In this study, BBS was utilized as a primary outcome measure for evaluating balance ability; however, the results did not indicate statistically significant improvements. Several factors may contribute to this outcome. First, the small sample sizes in studies reporting BBS outcomes may have limited the statistical power, thereby reducing the likelihood of detecting significant effects even in the presence of genuine balance improvements. Second, the intervention itself may have had a relatively modest impact on balance function compared to its effects on upper limb motor ability or other functional domains. Finally, the BBS relies on assessor judgment, which may be influenced by variability in assessment conditions, patient status, and inter-rater differences, potentially contributing to inconsistent results.

Although this study has demonstrated the relative advantages of tDCS combined with various rehabilitation interventions in improving limb dysfunction among PSSS, the practical implementation of these interventions still faces several challenges. Firstly, most existing studies have been conducted in well-equipped specialized hospitals or rehabilitation centers, whereas primary healthcare institutions may lack adequate infrastructure, equipment investment, and trained personnel for implementation and maintenance. Secondly, data on cost-effectiveness remain limited, making it difficult to determine the economic feasibility of these approaches in resource-constrained settings. Lastly, regional disparities in the distribution of rehabilitation resources and technological capabilities may further impede the widespread adoption of these strategies. Therefore, how to balance clinical efficacy and economic costs in real-world practice, and how to tailor these interventions to suit primary care settings, remain critical issues to be addressed. Future efforts should focus on conducting multicenter studies, health economic evaluations, and implementation research to assess the feasibility of these interventions under primary care conditions and to address potential barriers such as equipment procurement and workforce training.

This study has several limitations. Due to the lack of detailed information on stroke stages and the degree of motor dysfunction in most of the original studies, although our network meta-analysis provides relative efficacy for each intervention based on the existing evidence, these factors may lead to significant clinical heterogeneity. This study primarily relied on various clinical assessment scales (e.g., FMA, ARAT, BBS) to evaluate intervention outcomes. While these tools are widely used in clinical practice and offer considerable ease of use and acceptable reliability, they are inherently subjective, as they depend on observer ratings and patients’ self-performance. In contrast, objective indicators such as neuroimaging (e.g., MRI, fMRI) and electroencephalography (EEG) can more directly reflect changes in brain function and structure. Future research should consider integrating neuroimaging, EEG, or other electrophysiological data into study designs to provide more robust scientific evidence for the clinical application of these interventions. Due to insufficient reporting of treatment dosage parameters in combined intervention protocols across some included studies, we were unable to perform more comprehensive dose–response or subgroup analyses. This limitation may constrain the interpretation of intensity-efficacy relationships in our findings. Some studies have defects such as failure to strictly implement blinding or inconsistent procedures during measurements, which may introduce bias in the outcome measures and potentially lead to an overestimation of the efficacy of tDCS or combined tDCS treatment protocols. Therefore, readers should exercise caution when selecting intervention protocols. After excluding studies with high risk of bias and reanalyzing the data, no significant changes in the results were observed, which suggests that the findings of this study are stable.

## 5 Conclusion

BCIT+tDCS was identified as the optimal tDCS-based rehabilitation strategy for improving upper limb motor ability in PSSS, MT + tDCS was the most effective intervention for enhancing arm mobility, MT + tDCS was the best protocol for improving lower limb motor ability, while AM+tDCS was the best strategy for improving balance ability in PSSS. Furthermore, MT + tDCS was the optimal tDCS-based rehabilitation approach for treating upper limb motor dysfunction, whereas AM+tDCS was the most effective strategy for addressing lower limb motor dysfunction in PSSS. Future studies may focus on investigating the therapeutic effects of MT combined with tDCS on Berg Balance Scale score in PSSS, as well as the effects of AM combined with tDCS on Action Research Arm Test score, in order to further explore the therapeutic potential of these two intervention strategies.

## Data Availability

The data analyzed in this study is subject to the following licenses/restrictions: the datasets analysed during the current study are not publicly available but are available from the corresponding author on reasonable request. Requests to access these datasets should be directed to LG, liangguo@m.scnu.edu.cn.
