# Supplementary material for: Comparison of the effects of transcranial direct current stimulation combined with different rehabilitation interventions on motor function in people suffering from stroke-related symptoms: a systematic review and network meta-analysis
Source: Front Neurol. 2025 Jun 4;16:1586685. doi: 10.3389/fneur.2025.1586685 (PMC12173876; doi:10.3389/fneur.2025.1586685)
Supplement: Supplementary file 1 [file Table_1.docx]

**PubMed**

**#1** " transcranial direct current stimulation "[Mesh]

**#2** tDCS OR Anodal Stimulation Transcranial Direct Current Stimulation OR Anodal Stimulation tDCS* OR Cathodal Stimulation Transcranial Direct Current Stimulation OR Cathodal Stimulation tDCS* OR Transcranial Alternating Current Stimulation OR Transcranial Random Noise Stimulation OR Repetitive Transcranial Electrical Stimulation OR Transcranial Electrical Stimulation* [Title/Abstract]

**#3** #1 OR #2

**#4** " Stroke "[Mesh]

**#5** Stroke* OR Cerebrovascular Accident* OR CVA* OR Brain Vascular Accident* OR Apoplex* OR acute cerebrovascular lesion OR acute focal cerebral vasculopathy OR brain accident OR brain attack OR brain blood flow disturbance OR brain insult* OR cerebral insult OR cerebrovascular arrest OR cerebrovascular failure OR cerebrovascular injury OR cerebrovascular insufficiency OR cerebrovascular insult OR cerebrum vascular accident OR insultus cerebralis OR ischaemic seizure OR ischemic seizure [Title/Abstract]

**#6** #4 OR #5

**#7** Random* [Title/Abstract]

**#8** #3 AND #6 AND #7

**Cochrane Library**

**#1** MeSH descriptor: [transcranial direct current stimulation] explode all trees

**#2** tDCS OR Anodal Stimulation Transcranial Direct Current Stimulation OR Anodal Stimulation tDCS* OR Cathodal Stimulation Transcranial Direct Current Stimulation OR Cathodal Stimulation tDCS* OR Transcranial Alternating Current Stimulation OR Transcranial Random Noise Stimulation OR Repetitive Transcranial Electrical Stimulation OR Transcranial Electrical Stimulation* :ti,ab,kw

**#3** #1 OR #2

**#4** MeSH descriptor: [Stroke] explode all trees

**#5** Stroke* OR Cerebrovascular Accident* OR CVA* OR Brain Vascular Accident* OR Apoplex* OR acute cerebrovascular lesion OR acute focal cerebral vasculopathy OR brain accident OR brain attack OR brain blood flow disturbance OR brain insult* OR cerebral insult OR cerebrovascular arrest OR cerebrovascular failure OR cerebrovascular injury OR cerebrovascular insufficiency OR cerebrovascular insult OR cerebrum vascular accident OR insultus cerebralis OR ischaemic seizure OR ischemic seizure :ti,ab,kw

**#6** #4 OR #5

**#7** Random* :ti,ab,kw

**#10** #3 AND #6 AND #7

**Web of Science**

TS=(transcranial direct current stimulation OR tDCS OR Anodal Stimulation Transcranial Direct Current Stimulation OR Anodal Stimulation tDCS* OR Cathodal Stimulation Transcranial Direct Current Stimulation OR Cathodal Stimulation tDCS* OR Transcranial Alternating Current Stimulation OR Transcranial Random Noise Stimulation OR Repetitive Transcranial Electrical Stimulation OR Transcranial Electrical Stimulation*) AND TS=(Stroke* OR Cerebrovascular Accident* OR CVA* OR Brain Vascular Accident* OR “Apoplex*” OR “acute cerebrovascular lesion” OR “acute focal cerebral vasculopathy” OR brain accident OR brain attack OR brain blood flow disturbance OR brain insult* OR cerebral insult OR cerebrovascular arrest OR cerebrovascular failure OR cerebrovascular injury OR cerebrovascular insufficiency OR cerebrovascular insult OR cerebrum vascular accident OR insultus cerebralis OR ischaemic seizure OR ischemic seizure) AND TS=(Random*)

**Embase**

**#1** 'transcranial direct current stimulation'/exp OR 'tDCS':ab,ti OR 'Anodal Stimulation Transcranial Direct Current Stimulation':ab,ti OR 'Anodal Stimulation tDCS*':ab,ti OR 'Cathodal Stimulation Transcranial Direct Current Stimulation':ab,ti OR 'Cathodal Stimulation tDCS*':ab,ti OR 'Transcranial Alternating Current Stimulation':ab,ti OR ' Transcranial Random Noise Stimulation':ab,ti OR ' Repetitive Transcranial Electrical Stimulation':ab,ti OR 'Transcranial Electrical Stimulation*':ab,ti

**#2** 'cerebrovascular accident'/exp OR stroke*:ab,ti OR 'cerebrovascular accident*':ab,ti OR cva*:ab,ti OR 'brain vascular accident*':ab,ti OR 'apoplex*':ab,ti OR 'acute cerebrovascular lesion':ab,ti OR 'acute focal cerebral vasculopathy':ab,ti OR 'brain accident':ab,ti OR 'brain attack':ab,ti OR 'brain blood flow disturbance':ab,ti OR 'brain insult*':ab,ti OR 'cerebral insult':ab,ti OR 'cerebrovascular arrest':ab,ti OR 'cerebrovascular failure':ab,ti OR 'cerebrovascular injury':ab,ti OR 'cerebrovascular insufficiency':ab,ti OR 'cerebrovascular insult':ab,ti OR 'cerebrum vascular accident':ab,ti OR 'insultus cerebralis':ab,ti OR 'ischaemic seizure':ab,ti OR 'ischemic seizure':ab,ti

**#3** random*:ab,ti

**#4** #1 AND #2 AND #3

**VIP**

（经颅直流电刺激OR经颅直流电 OR tDCS）AND（脑卒中OR卒中OR脑血管疾病OR中风OR脑血管中风OR脑血管意外OR偏瘫）AND（随机）

**CNKI**

（经颅直流电刺激OR经颅直流电 OR tDCS）AND（脑卒中OR卒中OR脑血管疾病OR中风OR脑血管中风OR脑血管意外OR偏瘫）AND（随机）

**Wanfang**

（经颅直流电刺激OR经颅直流电 OR tDCS）AND（脑卒中OR卒中OR脑血管疾病OR中风OR脑血管中风OR脑血管意外OR偏瘫）AND（随机）

**SinoMed**

**#1** "经颅直流电刺激"[不加权:扩展]

**#2** "卒中"[不加权:扩展]

**#3** ( "脑卒中"[常用字段:智能] OR "卒中"[常用字段:智能] OR "脑血管疾病中风"[常用字段:智能] OR "脑血管中风"[常用字段:智能] OR "脑血管意外"[常用字段:智能] OR "偏瘫"[常用字段:智能])

**#4** ("经颅直流电刺激"[常用字段:智能] OR "经颅直流电"[常用字段:智能] OR "tDCS"[常用字段:智能])

**#5** "随机"[摘要:智能]

**#6** (#4) OR (#1)

**#7** (#3) OR (#2)

**#8** (#7) AND (#6) AND (#5)
