# Supplementary material for: Comparison of the effects of transcranial direct current stimulation combined with different rehabilitation interventions on motor function in people suffering from stroke-related symptoms: a systematic review and network meta-analysis
Source: Front Neurol. 2025 Jun 4;16:1586685. doi: 10.3389/fneur.2025.1586685 (PMC12173876; doi:10.3389/fneur.2025.1586685)
Supplement: Supplementary file 2 [file Table_2.DOCX]

**Supplementary Table** Summary Table of tDCS Parameters

| NO | Author | Year | tDCS Current Intensity | Single-session tDCS Duration | tDCS Intervention Period |
| --- | --- | --- | --- | --- | --- |
| 1 | Che XW | 2017 | 1.5mA | 40min | Once daily, five days per week, for a duration of four weeks |
| 2 | Chen HB | 2021 | 2mA | 20min | Once daily for twelve consecutive days |
| 3 | Chen H | 2020 | 2mA | 20min | Once daily, six days per week, for a duration of four weeks |
| 4 | Chen TT | 2023 | 2mA | 20min | Once daily, five days per week, for a duration of six weeks |
| 5 | Chen Y | 2022 | 2mA | 20min | Once daily, six days per week, for a duration of four weeks |
| 6 | Cheng P | 2015 | 1.2mA | 20min | Once daily, six days per week, for a duration of six weeks |
| 7 | Cheng XX | 2024 | 1.5mA | 30min | Five sessions per week for four consecutive weeks |
| 8 | Cui C | 2021 | 1mA | 30min | Once daily, five days per week, for a duration of four weeks |
| 9 | Deng R | 2024 | 2mA | 20min | Once daily, five days per week, for a duration of eight weeks |
| 10 | Dong K | 2021 | 2mA | 30min | Five sessions per week for two consecutive weeks |
| 11 | Feng CW | 2023 | 2mA | 20min | Once daily for four consecutive weeks |
| 12 | Gao L | 2024 | 2mA | 20min | Once daily, five days per week, for a duration of four weeks |
| 13 | Gao Z | 2021 | 2mA | 20min | Once daily, six days per week, for a duration of four weeks |
| 14 | Gao Z | 2023 | 2mA | 20min | Once daily, five days per week, for a duration of sixweeks |
| 15 | Han X | 2023 | 1mA | 20min | Once daily, five days per week, for a duration of four weeks |
| 16 | Hu HL | 2023 | 1.2mA | 20min | Five sessions per week for four consecutive weeks |
| 17 | Huang Y | 2023 | 1.4mA | 20min | Five sessions per week for four consecutive weeks |
| 18 | Jiang Y | 2020 | 1mA-1.5mA | 20min | Five sessions per week for two consecutive weeks |
| 19 | Jin J | 2019 | 2mA | 20min | Once daily, five days per week, for a duration of eight weeks |
| 20 | Jin MY | 2020 | 1mA-2mA | 30min | Six sessions per week for eight consecutive weeks |
| 21 | Li XL | 2021 | 2mA | 20min | Once daily, six days per week, for a duration of four weeks |
| 22 | Li YB | 2019 | 2mA | 20min | Once daily, six days per week, for a duration of four weeks |
| 23 | Liu LS | 2019 | 2mA | 20min | Twice daily, five days per week, for a duration of six weeks |
| 24 | Liu Y | 2023 | 1.6mA | 20min | Once daily, five days per week, for a duration of four weeks |
| 25 | Liu YW | 2020 | 2mA | 20min | Once daily, five days per week, for a duration of four weeks |
| 26 | Long SY | 2024 | 1.2mA | 20min | Five sessions per week for four consecutive weeks |
| 27 | Pan AH | 2023 | 1.5mA | 30min | Five sessions per week for four consecutive weeks |
| 28 | Qi YS | 2023 | 1mA-2mA | 20min | Once daily, five days per week, for a duration of four weeks |
| 29 | Qu F | 2024 | 2mA | 20min | Once daily, five days per week, for a duration of two weeks |
| 30 | Ren SS | 2023 | 2mA | 20min | Once daily, six days per week, for a duration of four weeks |
| 31 | Song DW | 2024 | 1mA | 20min | Once daily for two consecutive weeks |
| 32 | Sun FB | 2023 | 2mA | 20min | Once daily, five days per week, for a duration of six weeks |
| 33 | Tu M | 2021 | 2mA | 20min | Once daily for four consecutive weeks |
| 34 | Wang C | 2021 | 1mA | 20min | Once daily for eight consecutive weeks |
| 35 | Wang CY | 2023 | 1mA | 20min | Once daily, five days per week, for a duration of four weeks |
| 36 | Wang HB | 2023 | 2mA | 20min | Once daily, six days per week, for a duration of eight weeks |
| 37 | Wang HY | 2023 | 1.75mA | 20min | Five sessions per week for four consecutive weeks |
| 38 | Wang Y | 2021 | 2mA | 20min | Once daily, five days per week, for a duration of four weeks |
| 39 | Yin Y | 2015 | 1mA | 20min | Once daily, five days per week, for a duration of four weeks |
| 40 | Zhang SS | 2022 | 1.2mA | 20min | Five sessions per week for four consecutive weeks |
| 41 | Zhang Y | 2019 | 2mA | 20min-30min | Once daily, six days per week, for a duration of four weeks |
| 42 | Wang W | 2021 | 1.4mA | 20min | Twice daily, five days per week, for a duration of two weeks |
| 43 | Zhao F | 2021 | 1.4mA | 25min | Once daily, five days per week, for a duration of four weeks |
| 44 | Zhao JY | 2023 | 1mA | 20min | Five sessions per week for four consecutive weeks |
| 45 | Zheng CJ | 2019 | 1.5mA | 20min | Once daily, five days per week, for a duration of four weeks |
| 46 | Zheng S | 2020 | 1.5mA | 20min | Once daily for thirty-six consecutive days |
| 47 | Zhou YP | 2018 | 2mA | 20min | Once daily, six days per week, for a duration of eight weeks |
| 48 | Alisar | 2020 | 2mA | 30min | Once daily, five days per week, for a duration of three weeks |
| 49 | Cha | 2014 | 1mA | 20min | Once daily, five days per week, for a duration of four weeks |
| 50 | Cho | 2015 | 2mA | 20min | Three sessions per week for six consecutive weeks |
| 51 | Zeng | 2024 | 1.5mA | 20min | Once daily, five days per week, for a duration of four weeks |
| 52 | Gong | 2023 | 2mA | 20min | Once daily, five days per week, for a duration of four weeks |
| 53 | Lee | 2014 | 2mA | 20min | Five sessions per week for three consecutive weeks |
| 54 | Tedla | 2022 | 2mA | 20min | Four sessions per week for six consecutive weeks |
| 55 | Hsu | 2023 | 2mA | 20min | Twice daily, five days per week, for a duration of two weeks |
| 56 | Rabadi | 2020 | 1mA | 30min | Five sessions per week for two consecutive weeks |
| 57 | Qurat | 2023 | 2mA | 20min | Single session intervention |
| 58 | Li | 2024 | 2mA | 20min | Five sessions per week for four consecutive weeks |
| 59 | Lindenberg | 2010 | 1.5mA | 30min | Single session intervention |
| 60 | Llorens | 2021 | 2mA | 30min | Twenty-five sessions |
| 61 | Chang | 2015 | 2mA | 10min | Five sessions per week for two consecutive weeks |
| 62 | Duan | 2023 | 2mA | 20min | Once daily for twenty consecutive days |
| 63 | Youssef | 2023 | 2mA | 20min | Three sessions per week for four consecutive weeks |
| 64 | Toktas | 2024 | 2mA | 20min | Once daily, five days per week, for a duration of four weeks |
| 65 | Allman | 2016 | 1mA | 20min | Nine sessions |
| 66 | Dinesh | 2011 | 1mA | 30min | Once daily for five consecutive days |
| 67 | Fusco | 2014 | 1.5mA | 10min | Once daily, five days per week, for a duration of two weeks |
| 68 | Kim | 2010 | 2mA | 20min | Once daily, five days per week, for a duration of two weeks |
| 69 | Oveisgharan | 2017 | 2mA | 30min | Ten sessions over two weeks |
| 70 | Pinto | 2021 | 2mA-3mA | 30min | Twice daily, six days per week, for a duration of two weeks |
| 71 | Prathum | 2022 | 2mA | 20min | Three sessions per week for four consecutive weeks |
| 72 | Rossi | 2013 | 2mA | 20min | Once daily for five consecutive days |
| 73 | Lazzaro | 2014 | 2mA | 40min | Once daily for five consecutive days |
| 74 | Kim | 2024 | 2mA | 20min | Twelve sessions over four weeks |
